# Efficacy and Safety of Medicinal Plants Used in the Management of Diabetes Mellitus

**DOI:** 10.1155/2014/793035

**Published:** 2014-04-03

**Authors:** Musa T. Yakubu, Taofik O. Sunmonu, Francis B. Lewu, Anafi O. T. Ashafa, Femi J. Olorunniji, Mohamed Eddouks

**Affiliations:** ^1^Phytomedicine, Toxicology and Reproductive Biochemistry Research Laboratory, Department of Biochemistry, University of Ilorin, Ilorin 240003, Nigeria; ^2^Phytomedicine, Environmental Toxicology and Plant Biochemistry Research Laboratory, Department of Biological Sciences, Al-Hikmah University, Ilorin 240001, Nigeria; ^3^Department of Agriculture, Faculty of Applied Sciences, Cape Peninsula University of Technology, Wellington Campus, Wellington, Western Cape 7655, South Africa; ^4^Department of Plant Sciences, University of the Free State, Qwaqwa Campus, Phuthaditjhaba 9866, South Africa; ^5^Institute of Molecular, Cell, and System Biology, College of Medical, Veterinary, and Life Sciences, University of Glasgow, Glasgow G12 8QQ, UK; ^6^Faculty of Sciences and Techniques Errachidia, Moulay Ismail University, 52000 Errachidia, Morocco

This special issue edited by a team of five renowned scholars led by an Associate Professor of Biochemistry, M. T. Yakubu, Ph.D. (Lead Guest Editor), and other Guest Editors that included Professor T. O. Sunmonu, Dr. F. B. Lewu, Dr. A. O. T. Ashafa, Dr. Femi J. Olorunniji, and Professor Mohammed Eddouks features 15 original research papers covering various aspects of diabetes mellitus.

R. M. P. Gutierrez et al. in the paper entitled “*Ameliorative effect of hexane extract of Phalaris canariensis on high fat diet-induced obese and streptozotocin-induced diabetic mice*” reported the antiobesity effect of a hexane extract of* Phalaris canariensis *seed in high-fat diet and streptozotocin-induced diabetic mice. M. T. Sultan et al. in the paper entitled “*Nigella sativa fixed and essential oil supplementation modulates hyperglycemia and allied complications in streptozotocin-induced diabetes mellitus*” described the modulatory effects of* Nigella sativa* fixed and essential oil supplementation on hyperglycemia and allied complications in streptozotocin-induced diabetes mellitus. In a related study, M. I. Kazeem et al. in the paper entitled “*Protective effect of free and bound polyphenol extracts from ginger (Zingiber officinale Roscoe) on the hepatic antioxidant and some carbohydrate metabolizing enzymes of streptozotocin-induced diabetic rats*” evaluated the protective effect of free and bound polyphenol extracts from ginger (*Zingiber officinale* Roscoe) on the hepatic antioxidant and some carbohydrate metabolizing enzymes of streptozotocin-induced diabetic rats. P.-G. Cheng et al. in the paper entitled “*Polysaccharides-rich extract of Ganoderma lucidum (M.A. Curtis:Fr.) P. Karst accelerates wound healing in streptozotocin-induced diabetic rats*” evaluated the wound healing activity of the hot aqueous extract of* Ganoderma lucidum* in streptozotocin-induced diabetic rats. The work reported by da A. A. Rocha et al. in the paper entitled “*Lectin from Crataeva tapia bark improves tissue damages and plasma hyperglycemia in alloxan-induced diabetic mice*” revealed that lectin extracted from* Crataeva tapia* bark resulted in improvement of tissue damage and plasma hyperglycemia in alloxan-induced diabetic mice.

J. Gu et al. in the paper entitled “*A drug-target network-based approach to evaluate the efficacy of medicinal plants for type II diabetes mellitus*” used drug-target network-based approach to evaluate the efficacy of medicinal plants for type II diabetes mellitus. The study reported by M. M. Zainudin et al. in the paper entitled “*Does oral ingestion of Piper sarmentosum cause toxicity in experimental animals?*” investigated the potential toxicity of oral administration of an antidiabetic plant,* Piper sarmentosum*, in experimental animals. P. V. Rao et al. in the paper entitled “*Rhinacanthus nasutus improves the levels of liver carbohydrate, protein, glycogen, and liver markers in streptozotocin-induced diabetic rats*” showed that treatment of streptozotocin-induced diabetic rats with* Rhinacanthus nasutus* resulted in improvement in the levels of liver carbohydrate, protein, glycogen, and other hepatic markers. Y. Xiao et al. in the paper entitled “*The effect of Chinese herbal medicine on albuminuria levels in patients with diabetic nephropathy: a systematic review and meta-analysis*” presented a systematic review and meta-analysis of the effect of Chinese herbal medicine on albuminuria levels in patients with diabetic nephropathy. J. Wu et al. in the paper entitled “*Renal protective role of Xiexin decoction with multiple active ingredients involves inhibition of inflammation through downregulation of the nuclear factor-kB pathway in diabetic rats*” presented evidence to suggest that the inhibition of inflammation through downregulation of the nuclear factor-k*β* pathway in diabetic rats is the mechanism by which Xiexin decoction with multiple active ingredients exhibits its renal protective role. The paper by L. Zhou et al. in the paper entitled “*Hu-Lu-Ba-Wan attenuates diabetic nephropathy in type 2 diabetic rats through PKC-*α*/NADPH oxidase signaling pathway*” reported the attenuation of diabetic nephropathy in type 2 diabetic rats through PKC-*α*/NADPH oxidase signaling pathway by Hu-Lu-Ba-Wan. The evaluation of antidiabetic activity and associated toxicity of* Artemisia afra* aqueous extract in Wistar rats was reported by T. O. Sunmonu and A. J. Afolayan in the paper entitled “*Evaluation of antidiabetic activity and associated toxicity of Artemisia afra aqueous extract in Wistar rats.*”* N. A. Ishak et al. in the paper entitled* “*Antidiabetic and hypolipidemic activities of Curculigo latifolia fruit: root extract in high fat fed diet and low dose STZ induced diabetic rats*” evaluated the antidiabetic and hypolipidemic activities of* Curculigo latifolia* fruit: root extract in high-fat fed diet and low dose STZ-induced diabetic rats. The evaluation of the hypoglycemic properties of* Anacardium humile* aqueous extract was reported by M. A. Urzêda et al. in the paper entitled “*Evaluation of the hypoglycemic properties of Anacardium humile aqueous extract.*” Finally, the decrease of plasma glucose by* Hibiscus taiwanensis* in type-1-like diabetic rats was the focus of the study reported by L.-Y. Wang et al. in the paper entitled “*Decrease of plasma glucose by Hibiscus taiwanensis in type-1-like diabetic rats*.”

The variety of papers on diabetes featured in this special issue highlights the keen awareness of the biomedical community of the potential for exploiting medicinal plants in tackling this debilitating condition. Of particular interest is the large number of contributions from scientists working in developing countries. This level of interest should be encouraged. The gradual improvement in the level of prosperity in developing economies is being accompanied by a significant increase in reported cases of obesity, diabetes, and other metabolic disorders. It is appropriate that scientists and policy makers in these lands devote more resources to exploring newer ways of dealing with these conditions. It is hoped that the encouraging findings reported in this special issue will stimulate further interest in expanding and coordinating efforts on medicinal plants that have clinical potential in the management of diabetes.

